# Hierarchical multivariate covariance analysis of metabolic connectivity

**DOI:** 10.1038/jcbfm.2014.165

**Published:** 2014-10-08

**Authors:** Felix Carbonell, Arnaud Charil, Alex P Zijdenbos, Alan C Evans, Barry J Bedell, for the Alzheimer's Disease Neuroimaging Initiative

**Affiliations:** 1 Biospective Inc., Montreal, QC, Canada; 2 Montreal Neurological Institute, McGill University, Montreal, QC, Canada

**Keywords:** Alzheimer's disease, *β*-amyloid, covariance analysis, FDG PET, florbetapir PET, metabolic connectivity, mild cognitive impairment

## Abstract

Conventional brain connectivity analysis is typically based on the assessment of interregional correlations. Given that correlation coefficients are derived from both covariance and variance, group differences in covariance may be obscured by differences in the variance terms. To facilitate a comprehensive assessment of connectivity, we propose a unified statistical framework that interrogates the individual terms of the correlation coefficient. We have evaluated the utility of this method for metabolic connectivity analysis using [18F]2-fluoro-2-deoxyglucose (FDG) positron emission tomography (PET) data from the Alzheimer's Disease Neuroimaging Initiative (ADNI) study. As an illustrative example of the utility of this approach, we examined metabolic connectivity in angular gyrus and precuneus seed regions of mild cognitive impairment (MCI) subjects with low and high *β*-amyloid burdens. This new multivariate method allowed us to identify alterations in the metabolic connectome, which would not have been detected using classic seed-based correlation analysis. Ultimately, this novel approach should be extensible to brain network analysis and broadly applicable to other imaging modalities, such as functional magnetic resonance imaging (MRI).

## Introduction

Alterations in structural, functional, and metabolic connectivity are increasing recognized as key features of many neurologic and psychiatric diseases. Conventional brain connectivity analysis is typically based on the evaluation of interregional correlations.^1, 2, 3, 4, 5, 6, 7, 8^ A major limitation associated with this approach, however, is that correlation coefficients are derived from both the covariance and the variance of the data. As such, while group differences in covariance (connectivity) may exist, this information may not be captured by the correlation coefficient due to differences in the variance terms. It has been reported that several factors may affect the size of the Pearson correlation coefficient, thereby yielding misleading interpretations.^9^ These factors include the extent of variability in the data (e.g., range restriction^10^), differences in the shape of the data distrubutions, lack of linearity, outliers, characteristics of the sample, and measurement error.^9^ As such, the between-group comparisons of interregional correlations may be influenced by these factors. For instance, if the variance significantly differs across groups, then the interregional correlations tend to decrease for the group with higher variance. A potential source of methodological error leading to differences in group variances may occur when data have been collected in a multicenter study and the groups are not appropriately balanced at the different sites. Further, the variance in connectivity analysis is related not only to methodolgical issues, but also to biologic sources, including interindividual differences in functional neuroanatomy, variability in the regional distribution of activity, and differential levels of hormonal factors, stress, and anxiety.^11^

Proper assessment of connectivity, therefore, requires a unified statistical framework that interrogates the individual terms of the correlation coefficient. To this end, we propose a novel, hierarchical, multivariate approach based on between-group comparisons of 2 × 2 variance–covariance matrices. Our approach is based on hierarchical testing of between-group differences in the correlation coefficient and its component differences arising from the covariance and variance of the data. This multivariate approach is particularly useful when between-group differences in connectivity patterns are subtle and cannot be detected using more traditional univariate approaches, such as seed-based correlations. In addition, this new method has the advantage of including the conventional analysis of correlation coefficients as a particular case.

Metabolic connectivity examines the relationship between glucose metabolism in different brain regions. In their seminal paper, Horwitz *et al.*^1^ described across-subject metabolic correlations between brain regions based on [18F]2-fluoro-2-deoxyglucose (FDG) positron emission tomography (PET) scans, and identified strong correlations between homotopic regions and homologous regions in the left and right hemispheres. This work showed that correlation patterns based on FDG PET were consistent with anatomic and functional data, thereby validating the assertion that functional interactions reflect underlying anatomic pathways.^1^ The earliest studies of metabolic connectivity in Alzheimer's disease (AD) patients were performed by Metter *et al.*^12^ and Horwitz *et al.*^13^ who showed that AD patients had fewer consistent partial correlation coefficients compared with healthy control subjects. A more formal statistical approach, called SSM (Scaled Subprofile Model), was proposed by Moeller *et al.*^14^ for the study of brain metabolic networks. On the basis of Principal Components Analysis (PCA), SSM represents regional glucose metabolism profiles as a combination of region-independent global effects, a group mean pattern, and a mosaic of interacting AD-related networks.^15,16^ Metabolic connectivity approaches based on multivariate data decomposition techniques (e.g., PCA and Independent Components Analysis) have also been proposed in recent years.^17, 18, 19, 20, 21^ Several studies have systematically explored whole-brain metabolic connectivity patterns using seed-based correlation analyses. Under the basic general linear model assumptions, the seed-based correlation approach uses the mean FDG uptake in a seed region as a covariate-of-interest in a classic statistical parametric mapping analysis, and correlates it with all other voxels over the entire brain. Using this approach, Mosconi *et al.*^2^ performed whole brain, voxel-based correlation analyses to assess functional interactions of the entorhinal cortex. More recently, Lee *et al.*^5^ extended this approach for the establishment of normative data for interregional metabolic connectivity. Our group has recently explored the relationship between *β*-amyloid burden and metabolic connectivity patterns at early stages of AD using a seed-based, metabolic correlation analysis.^22^ Specifically, we have shown that alterations in metabolic connectivity are related to the presence of fibrillar, *β*-amyloid deposits and are not a function of genotype. While our previous analysis^22^ clearly showed reduced metabolic correlations as a function of increased amyloid burden, the seed-based correlations for several ‘hub' regions, such as the precuneus, a key node of the default-mode network (DMN), unexpectedly did not show any significant group differences in metabolic connectivity. Given that previous studies have shown aberrant functional connectivity, based on resting-state functional magnetic resonance imaging (MRI) data, between posterior cingulate/precuneus and other brain regions as a function of *β*-amyloid positivity,^23^ we sought to further interrogate metabolic connectivity through a more sophisticated analysis strategy that could better capture the relationship between disease-related alterations in the functional and metabolic connectomes.

In this paper, we evaluate the utility of the proposed HMC (Hierarchical Multivariate Covariance) analysis using FDG PET data from the Alzheimer's Disease Neuroimaging Initiative (ADNI) study. Our main objective is to show that traditional univariate approaches to metabolic connectivity, such as seed-based correlation analysis, can fail to reveal important interregional relationships, while the HMC analysis facilitates a more thorough exploration of the metabolic network architecture. To this end, we have applied this HMC analysis to FDG PET data from mild cognitive impairment (MCI) subjects with low and high *β*-amyloid burdens, and we provide results from two illustrative cortical hub regions (angular gyrus and precuneus). These results reveal important new information concerning progressive metabolic disconnection within the DMN of MCI patients.

## Materials and methods

### Subjects and Image Acquisition

Data used in the preparation of this article were obtained from the ADNI database (http://adni.loni.usc.edu). The ADNI was launched in 2003 by the NIA (National Institute on Aging), the NIBIB (National Institute of Biomedical Imaging and Bioengineering), the FDA (Food and Drug Administration), private pharmaceutical companies, and non-profit organizations, as a $60 million, 5-year public private partnership. The primary goal of ADNI has been to test whether serial MRI, PET, other biologic markers, and clinical and neuropsychological assessment can be combined to measure the progression of MCI and AD. Determination of sensitive and specific markers of very early AD progression is intended to aid researchers and clinicians to develop new treatments and monitor their effectiveness, as well as lessen the time and cost of clinical trials. The ADNI is the result of efforts of many coinvestigators from a broad range of academic institutions and private corporations, and subjects have been recruited from over 50 sites across the United States and Canada. The initial goal of ADNI was to recruit 800 subjects, but ADNI has been followed by ADNI-GO and ADNI-2. To date, these three protocols have recruited over 1,500 adults, ages 55 to 90, to participate in the research, consisting of cognitively normal older individuals, people with early or late MCI, and people with early AD. The follow-up duration of each group is specified in the protocols for ADNI-1, ADNI-2, and ADNI-GO. Subjects originally recruited for ADNI-1 and ADNI-GO had the option to be followed in ADNI-2. For up-to-date information, see www.adni-info.org.

The cohort for this study consisted of 276 ADNI participants diagnosed with MCI who had available [18F]florbetapir PET, FDG PET, and 3D T1-weighted anatomic MRI scans. A detailed description of the MRI and PET image acquisition protocols can be found at http://adni.loni.usc.edu/methods. This study was approved by the Institutional Review Boards of all of the participating institutions. Informed written consent was obtained from all participants at each site.

### Image Processing

All MRI and PET images were processed using the PIANO software package (Biospective Inc., Montreal, QC, Canada). T1-weighted MRI volumes underwent image nonuniformity correction using the N3 algorithm,^24^ brain masking, linear spatial normalization utilizing a 9-parameter affine transformation, and nonlinear spatial normalization^25^ to map individual images from native coordinate space to MNI reference space using a customized, anatomic MRI template derived from ADNI subjects. The resulting image volumes were segmented into gray matter, white matter, and cerebrospinal fluid using an artificial neural network classifier^26^ and partial volume estimation.^27^ The gray matter density map for each subject was transformed to the same final spatial resolution (i.e., resampled to the same voxel size and spatially smoothed) as the FDG PET data to account for confounding effects of atrophy in the statistical model. The cerebral mid-cortical surface (i.e., the midpoint between the pia and the white matter) for each hemisphere was extracted to allow for surface projection of PET data using a modified version of the CLASP algorithm.^28^

The florbetapir PET and FDG images underwent several preprocessing steps, including frame-to-frame linear motion correction, smoothing using a scanner-specific blurring kernel, and concatenation of dynamic frames into a static image. The PET volumes were linearly registered to the subject T1-weighted MRI and, subsequently, spatially normalized to reference space using the nonlinear transformations derived from the anatomic MRI registration. Voxelwise standardized uptake value ratio (SUVR) maps were generated from both florbetapir and FDG PET using full cerebellum and pons as the reference regions, respectively. The cortical SUVR measures were projected onto the cortical surface, and the data from each subject were mapped to a customized surface template by non-rigid 2D surface registration for visualization purposes.^29^

### Subject Classification

The mean [18F]florbetapir SUVR was computed from a composite bilateral region-of-interest (ROI) comprised of the precuneus, posterior cingulate, and medial frontal cortex, for each subject (SUVR_ROI_). A Regularized Discriminant Analysis^30^ was performed to determine the optimal threshold to separate subjects into two distinct classes based on individual SUVR_ROI_ measurements, as previously described.^22^ Subjects with an SUVR_ROI_ value of ≤1.22 were designated as Amyloid-Low (A*β*_L_), and this group consisted of 139 subjects with an average SUVR_ROI_ value of 1.03±0.08 (mean±s.d.). The remaining 137 subjects, with average SUVR_ROI_ values of 1.50±0.16, were classified as Amyloid-High (A*β*_H_).

### Hierarchical Multivariate Covariance analysis

A voxelwise, two-way analysis-of-covariance model that included gender and apolipoprotein E *ɛ*4 genotype as categorical variables, age and mini-mental state exam as global covariates, as well as gray matter density as a voxelwise covariate was fitted to the FDG SUVR data. The apolipoprotein E ɛ4 status was included to eliminate any potential, misleading differences due to genotype, while gray matter density was included with the purpose of minimizing potential confounds related to intersubject differences in brain atrophy.^31,32^ The FDG SUVR residuals resulting from this model were used for the hierarchical multivariate covariance analysis.

The average FDG SUVR values were computed within the angular gyrus and precuneus ‘seed regions' for each subject. These two seeds were selected based on our previous metabolic connectivity analysis.^22^ In particular, these seeds were identified as ‘hubs' (i.e., highly correlated regions) in each of the two groups. The hub locations were determined as the local maximum of our previously defined metabolic connectivity strength measure. The seed regions consisted of a 6-mm-radius sphere centered at the following MNI space coordinates: (56, −48, 34) for the right angular gyrus and (8, −56, 40) for the right precuneus.

For each group of subjects, variance maps, *var(v)*, and seed-based covariance maps, *cov(s,v)*, were generated by covarying the average FDG SUVR within the seed region, *s,* with the FDG SUVR from all other voxels, *v,* over the entire brain. In contrast to seed-based cross-correlation maps *r(s,v)*, these seed-based covariance maps *cov(s,v)* are derived from the covariance measure, rather than the Pearson correlation coefficient. Effectively, seed-based cross-correlation maps can be interpreted as voxelwise, variance-normalized versions of the seed-based covariance maps. Seed-based covariance analysis involves between-group comparison of the corresponding 2 × 2 variance–covariance matrix, 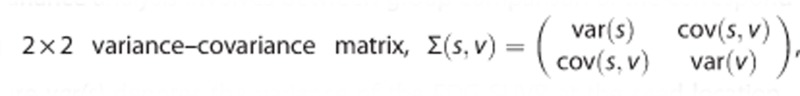 where *var(s)* denotes the variance of the FDG SUVR at the seed location.

Between-group differences in the variance–covariance matrices, Σ(*s,v*), can arise from several conditions, specifically: (1) proportionality of the matrices, (2) different variances/covariance, and (3) different correlations. More formally, possible relations between two covariance matrices, Σ_1_(*s,v*) and Σ_2_(*s,v*), can be expressed as the following hypotheses:


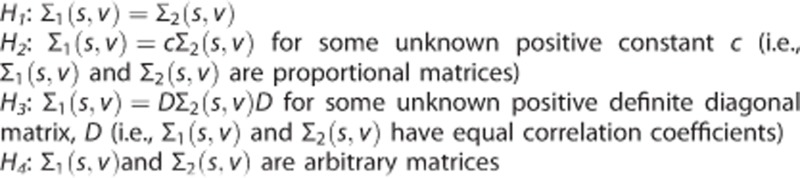


Hypothesis *H*_*1*_ is the classic null model for assessing equality between two different covariance patterns, Σ_1_(*s,v*) and Σ_2_(*s,v*), which is usually tested against the alternative model *H*_*4*_ to establish that no particular relationship exists between the two patterns. Hypothesis *H*_*2*_ is a null model for the case of proportional covariance patterns, which may occur in the presence of a global scaling factor affecting one of the two groups under study. Finally, hypothesis *H*_*3*_ covers the typical null model with expectation of equality in correlation coefficients, which has been the standard hypothesis in current metabolic seed-based correlation analysis.^5,7,22^

According to Seber,^33^ the classic method for testing *H*_*1*_ against *H*_*4*_ is based on a likelihood ratio test *T*_*0*_ (see definition in the Supplementary Material). Given that the hypothesis *H*_*i*_ is nested in the hypothesis *H*_*i+1*_ *(i=1, 2, 3),* Manly and Rayner^34^ showed that the likelihood ratio test, *T*_*0*_, can be hierarchically decomposed into three different test components, *T*_*1*_, *T*_*2*_, and *T*_*3*_, in such a way that *T*_*0*_*=T*_*1*_+*T*_*2*_ *+T*_*3*_ (see definitions of *T*_*1*_, *T*_*2*_, and *T*_*3*_ in the Supplementary Material). Moreover, each of these three components tests the hypothesis *H*_*i*_ against the hypothesis *H*_*i+1*_ *(i=1, 2, 3)* in a hierarchical manner. The *T*_*3*_ statistic tests the null hypothesis of the two groups having equal correlations (*H*_*3*_) against the alternative hypothesis of the two groups having arbitrary covariance matrices (implying unequal arbitrary correlation coefficients, *H*_*4*_). Since the hypothesis *H*_*2*_ is a particular case of the hypothesis *H*_*3*_ (i.e., proportional covariance matrices imply equal correlations), the null hypothesis *H*_*2*_ can be tested (using the *T*_*2*_ statistic) against the alternative hypothesis *H*_*3.*_ Thus, rejection of *H*_*2*_ due to statistically significant, high values of *T*_*2*_ would imply having two groups with different variances and covariance terms, but equal correlation coefficients. Testing the hypothesis *H*_*2*_ against the hypothesis *H*_*3*_ is extremely useful to reveal subtle, seed-based metabolic connectivity patterns that are typically obscured either by a relative increase in data variability in one of the groups or by a cross-relationship between variability (variance) and covariability (covariance) between groups. Finally, the *T*_*1*_ statistic tests the null hypothesis of the two groups having equal covariance matrices (*H*_*1*_) against the alternative hypothesis of the two groups having proportional covariance matrices (*H*_*2*_).

Therefore, in our voxelwise hierarchical testing procedure, *T*_*3*_*(s,v)* is first tested to assess whether it is significantly large in some regions, *V*_*3*_. If this observation is the case, then between-group differences occur in the correlations *r(s,V*_*3*_) between the seed and the composite of brain regions denoted by *V*_*3*_. *T*_*2*_*(s,v)* is then assessed for significance at the remaining brain regions which do not include *V*_*3*_. If statistically significantly large values of *T*_*2*_*(s,v)* are obtained in some regions, *V*_*2*_, then correlations, *r(s, V*_*2*_), are assumed to be equal and between-group differences occur in either the covariance, *cov(s, V*_*2*_), or the variance, *var(V*_*2*_). Finally, *T*_*1*_*(s,v)* is assessed for significance at the remaining brain regions exclusive of *V*_*3*_ and *V*_*2*_. If *T*_*1*_*(s,v)* is statistically significant in a region *V*_*1*_, then the variance–covariance matrices Σ(*s,V*_1_) are assumed to be proportional across groups; otherwise, they are assumed to be equal in the remaining voxels. An issue that can affect interpretation is that test *T*_*2*_ does not discriminate between differences arising from the covariance, *cov(s, V*_*2*_), and differences coming from the variances, *var(V*_*2*_) and *var(s)*. To overcome this limitation, we have considered a parallel likelihood ratio test for the variance term. Although several tests have been proposed for between-group comparisons of the variance (e.g., Levene's test), we took advantage of the fact that the variance term can be considered as a one-dimensional variance–covariance matrix, and have tested differences in variance using the likelihood ratio test, *T*_*0*_, with one degree of freedom. Given that each of these *t-*statistics follows a *χ*^2^ distribution at each voxel *v* (see expressions for the corresponding degrees of freedom in Supplementary Material), we used the false discovery rate^35^ (FDR) procedure at *α*=0.05 to control for multiple comparisons.

## Results

Subject characteristics are reported in Table 1. Although the whole sample was not equally distributed between males and females, the association between gender and amyloid status was not statistically significant (*P=*0.79). The age of A*β*_H_ subjects (74.22±7.02) was significantly higher than that of A*β*_L_ subjects (71.47±8.60) (*P*=0.002). There was also a statistically significant main effect of amyloid status on mini-mental state exam (*P*<0.001).

**Figure 1:**
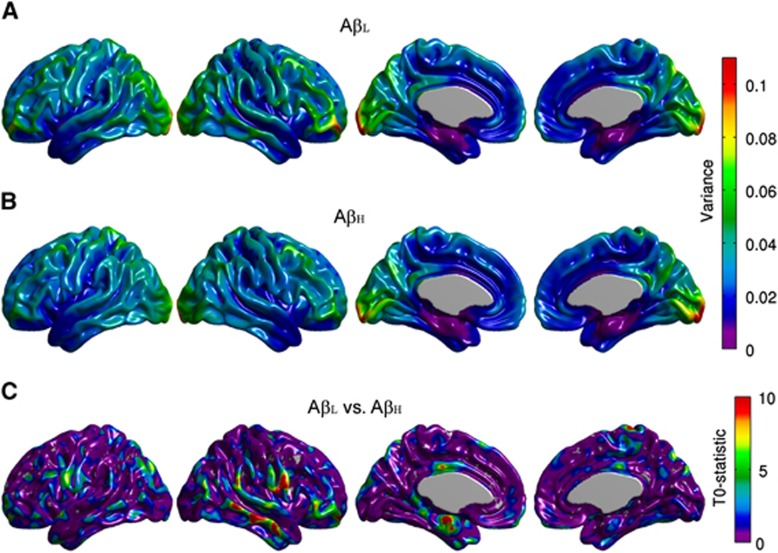
Representative surface views (*left-to-right:* left lateral, right lateral, left medial, and right medial) of the [18F]2-fluoro-2-deoxyglucose (FDG) standardized uptake value ratio (SUVR) variance maps for the Amyloid-Low (A*β*_L_) (**A**) and Amyloid-High (A*β*_H_) groups (**B**). Although not statistically significant, there are some small differences in variance in regions including the right middle temporal gyrus, right Rolandic operculum, and left entorhinal cortex (**C**).

The variance maps for the A*β*_L_ and A*β*_H_ groups are shown in Figure 1. Both groups show high across-subject variability in the occipital lobe. Figure 1C shows the *T*_*0*_-statistic map for the between-group comparison of the variance maps. Despite the presence of some small clusters of relatively high *T*_*0*_ values in the left entorhinal cortex, right middle/superior temporal gyrus, right pars orbitalis, and right Rolandic operculum, there were not any statistically significant differences. In particular, there were no statistically significant between-groups differences in variance at either the right angular gyrus seed (*T*_*0*_*=*1.815, *P*=0.17) or the right precuneus seed (*T*_*0*_=1.827, *P*=0.18)*.*

**Figure 2:**
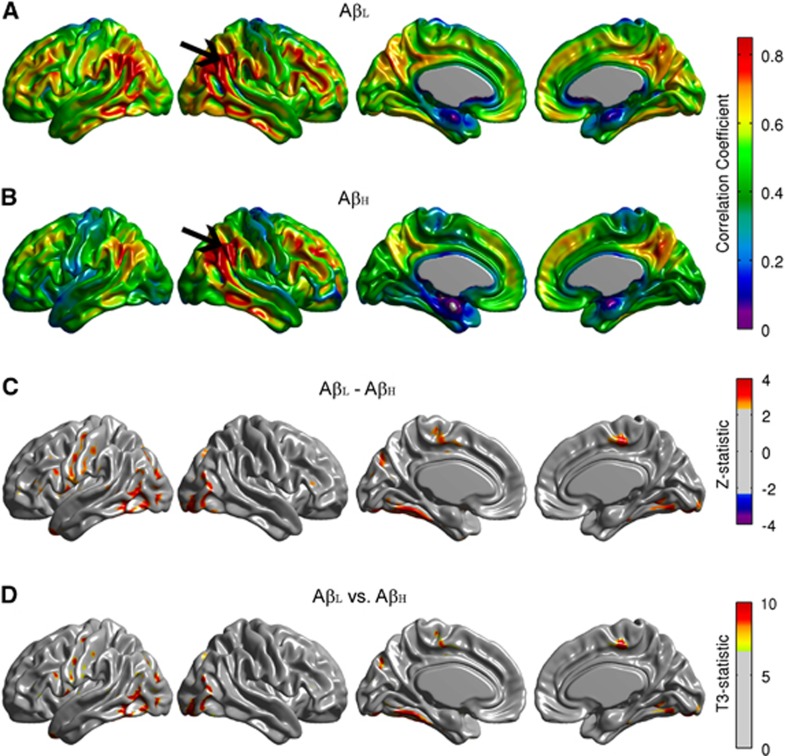
Seed-based correlation maps for the right angular gyrus. Maps are provided for the Amyloid-Low (A*β*_L_) group (**A**), Amyloid-High (A*β*_H_) group (**B**), as well as false discovery rate (FDR)-thresholded Z-statistic and *T*_3_-statistic for the A*β*_L_ versus A*β*_H_ group differences (**C,** **D**). The arrows indicate the seed region. Although *Z* and *T*_3_ are not mathematically equivalent, they show similar regions of significantly reduced correlations in the A*β*_H_ group, especially with the left fusiform gyrus, bilateral paracentral lobule, bilateral inferior frontal gyrus, and left precentral and postcentral gyri.

**Figure 3:**
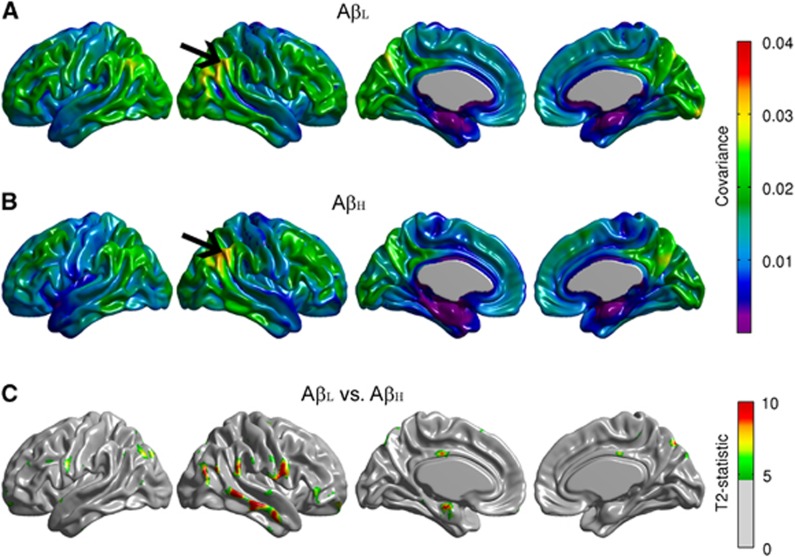
Seed-based covariance maps for the right angular gyrus. Maps are provided for the Amyloid-Low (A*β*_L_) group (**A**), Amyloid-High (A*β*_H_) group (**B**), as well as false discovery rate (FDR)-thresholded *T*_2_-statistic for the A*β*_L_ versus A*β*_H_ group comparison (**C**), which shows differences between the right angular gyrus and the right middle temporal gyrus, the right Rolandic operculum, and the left entorhinal cortex. These additional metabolic connectivity differences are likely produced by the small differences in variance observed in Figure 1.

Conventional seed-based correlation maps and the *T*_*3*_-statistic maps for the right angular gyrus are provided in Figure 2. The A*β*_H_ group showed reduced intrahemispheric and interhemispheric correlations compared with the A*β*_L_ group, especially between the seed and the left fusiform gyrus, bilateral paracentral lobule, bilateral occipital-temporal regions, and left precentral and postcentral gyri (Figure 2C). Despite the fact that the *T*_*3*_-statistic (Figure 2D) and the Z-test (Figure 2C) are not mathematically equivalent, they yield very similar results. Effectively, correlation analysis is a particular case of the generalized covariance analysis developed in this paper. The covariance maps for the right angular gryus are shown in Figure 3. The FDR-thresholded *T*_*2*_-statistic map resulting from the seed-based covariance analysis (Figure 3C) shows between-group metabolic connectivity differences between the right angular gyrus and small regions, including the right middle temporal gyrus, the right Rolandic operculum, and the left entorhinal cortex, which were not observed in the *T*_*3*_ map (Figure 2D). These additional metabolic connectivity differences are likely produced by the small differences in variance observed in Figure 1.

**Figure 4:**
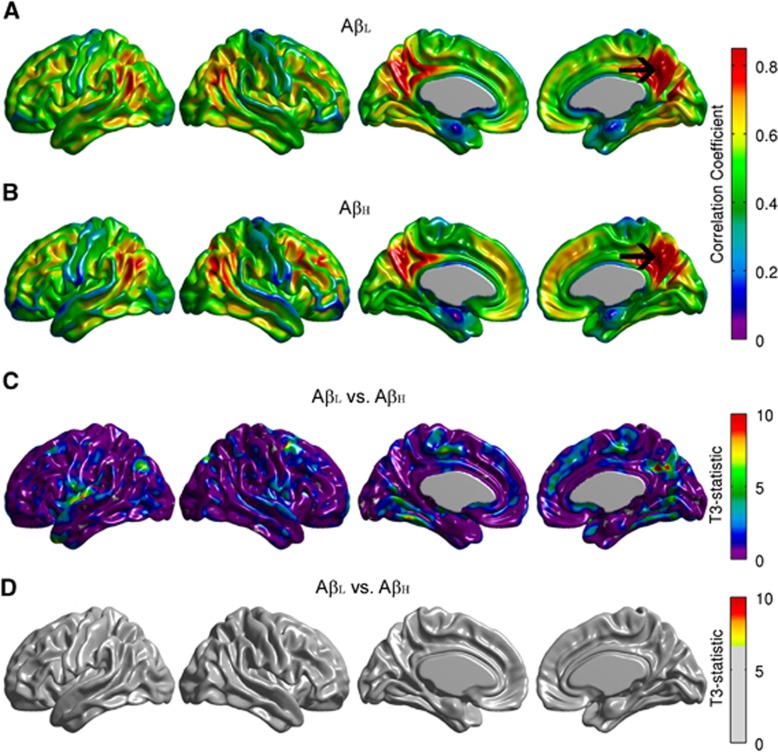
Seed-based correlation maps for the right precuneus. Maps are provided for the Amyloid-Low (A*β*_L_) group (**A**), Amyloid-High (A*β*_H_) group (**B**), as well as unthresholded and false discovery rate (FDR)-thresholded *T*_3_-statistic for the A*β*_L_ versus A*β*_H_ group comparison (**C**, **D**). The T_3_-map did not show any statistically significant between-groups differences in correlation (**D**).

**Figure 5:**
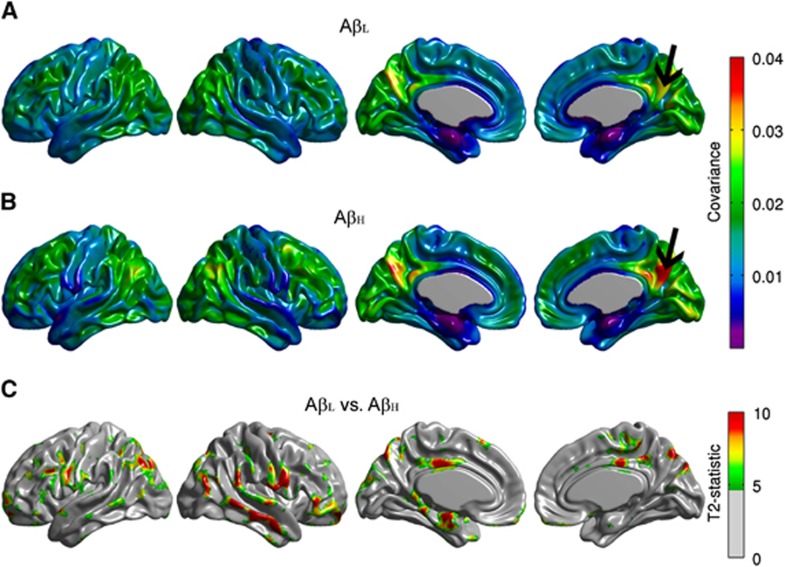
Seed-based covariance maps for the right precuneus. Maps are provided for the Amyloid-Low (A*β*_L_) group (**A**), Amyloid-High (A*β*_H_) group (**B**), as well as false discovery rate (FDR)-thresholded *T_2_*-statistic for the A*β*_L_ versus A*β*_H_ group comparison (**C**), which shows several regions of statistically significant differences, particularly between the precuneus and extended regions including right middle and superior temporal gyri, right pars orbitalis, left entorhinal cortex, bilateral Rolandic operculum, and bilateral posterior cingulate cortex.

Figure 4 shows the conventional seed-based correlation maps and the *T*_*3*_-statistic map resulting from the seed-based covariance analysis for the precuneus seed region. Note that no between-groups differences were observed in the correlation structure as a function of *β*-amyloid status (Figure 4D). In contrast, Figure 5C shows that the *T*_*2*_-statistic map produced several regions of statistically significant differences, particularly between the precuneus and extended cortical regions, including the right middle and superior temporal gyri, right pars orbitalis, left entorhinal cortex, bilateral Rolandic operculum, and bilateral posterior cingulate cortex. These findings provide evidence that, despite the correlation coefficients not being statistically significantly different between A*β*_L_ and A*β*_H_ groups, there were, in fact, metabolic connectivity differences. These differences could arise from either the covariance or the variance terms. While some of these differences are likely the result of differences in group variance terms (Figure 1C), not all of these differences can be simply explained by the variance given that the statistically significant regions in Figure 5C are more spatially extended than those of Figure 3C. In these regions, the detected connectivity is associated with the covariance. Interestingly, while the small between-group differences in variance observed in Figure 1 are not sufficient to result in the significant differences observed in Figure 5C, these differences counterbalance the covariance term in such a way to effectively eliminate differences in the seed-based correlation structure (Figure 4).

To assess the effects of high variance on the *T*_*2*_-statistic, we placed a seed in the right middle temporal gyrus, which is the region of highest between-group differences in variance (Figure 1). The resulting *T*_*2*_-statistic map (Supplementary Figure 1C) revealed particular regions (but not the whole cortex) of statistically significant connectivity differences, including bilateral inferior temporal gyrus, fusiform gyrus, and precuneus. This result is fully consistent with Figure 5, which showed that the *T*_*2*_ map relative to the right precuneus was statistically significant at the right middle temporal gyrus.

## Discussion

In this work, we have introduced a new statistical framework for connectivity analysis. As an illustrative example of the utility of this approach, we examined metabolic connectivity in MCI subjects with low and high *β*-amyloid burdens. This new, multivariate method allowed us to identify alterations in the metabolic connectome which would not have been detected using classic seed-based correlation analysis.

By explicitly examining the covariance, we were able to identify metabolic connectivity patterns that were not observed in our previous study,^22^ due to the relatively high variability in FDG SUVR measures across subjects. While not statistically significant, the A*β*_L_ group appeared to have a higher variability than the A*β*_H_ group in particular brain regions, such as the temporal–parietal areas. The relatively lower variability in the A*β*_H_ group may be explained by transition to a state of vulnerability to AD-related pathology, including *β*-amyloid deposition and greater cognitive deficits. Conversely, subjects in the more heterogeneous A*β*_L_ groups may be at different, earlier stages of disease progression. Our results are in concordance with those of Walhovd *et al.*^36^ who showed statistically significant differences, by Levene's test, in FDG PET variances between MCI and normal controls for several brain regions, including the precuneus and the posterior cingulate cortex.

In this particular study, we assessed the metabolic connectivity associated with the angular gyrus, the precuneus, and the middle temporal gyrus. The angular gyrus mediates language and semantic processing, as well as spatial attention and orientation, and is a key parietal node of the DMN. Our results are consistent with those reported by Jacobs *et al.*^37^ which implicated strong connectivity patterns between the angular gyrus and other brain areas as a driving factor for the involvement of the parietal lobe in AD. With respect to the precuneus, our results agree with those of Salmon *et al.*^19^ who showed that the precuneus was a core area in AD and found that it is associated with three different metabolic covariance networks, as determined by PCA. We were able to identify statistically significant differences between A*β*_L_ and A*β*_H_ groups for the precuneus based on covariance, but not based on correlation (Figures 4 and 5). The precuneus is a key hub of the DMN. Buckner *et al.*^38^ have shown that brain regions accumulating greater levels of *β*-amyloid correspond to functional hubs identified by increased functional connectivity with other brain regions in AD patients. Our new finding directly implicates *β*-amyloid accumulation in the progressive metabolic disconnection within the DMN of MCI patients. The increased sensitivity of our approach likely arises from increased intragroup variance in FDG PET signal that is better handled by covariance analysis than by correlation analysis. Indeed, the cross-relationship observed between variances and covariance in the two *β*-amyloid groups might lead to the false conclusion of a lack of connectivity disruption when solely using correlations coefficients. On the basis of these data, we can conclude that the intrinsic relationship between *β*-amyloid accumulation and metabolic connectivity is a clear pathologic feature of MCI/AD, and should be considered in conjunction with other pathologic features and imaging biomarkers, such as regional *β*-amyloid deposition and cerebral glucose hypometabolism. The novel data revealed by our HMC approach are consistent with the observations of Drzezga *et al*,^23^ which implicate the posterior cingulate/precuneus as a region highly susceptible to *β*-amyloid-related alterations in functional connectivity. The relationship between functional connectivity (based on the blood oxygen level-dependent signal) and metabolic connectivity (based on glucose uptake/metabolism) is currently poorly understood, and they cannot be presumed to be proxies of one another. It is conceivable that amyloid-related pathology may give rise to a decoupling of blood flow and metabolism such that they react differently to cognitive demand, exhibit a different disease time course, and respond differently to putative therapeutic intervention. It is, therefore, essential to examine the connectivity properties of both flow-related and metabolic markers. By properly assessing the covariance structure of FDG PET data, we have gleaned further insight into the effects of *β*-amyloid on cerebral metabolism and the apparent similarities to alterations in cerebrovascular physiology and neurovascular coupling. These intriguing results support future studies designed to examine connectivity patterns based on different modalities acquired from the same subjects.

From a methodological perspective, our method shares similarities to the approach employed by Salmon *et al.*^19^ and Walhovd *et al.*^36^ in that the statistical tests acted over covariances and variances, rather than over correlations. As such, in contrast to the classic seed-based correlation analysis, our multivariate approach allows for a proper comparison with PCA. The hierarchical *t*-tests described here facilitate comparisons with PCA networks computed by either covariance or correlation matrices. The *T*_*3*_-test is comparable to a PCA computed over correlation matrices, while the *T*_*2*_-test is comparable to a PCA based on covariance matrices. As such, our multivariate acts as a unified framework for combining statistical comparisons over connectivity structures arising simultaneously from covariances, variances, and correlations.

The multivariate approach introduced in this paper can be extended in several ways. While we have used the seed-based covariance approach for the comparison of two groups of subjects with different levels of cortical *β*-amyloid, our results can be readily extended to more than two groups. The expressions for the likelihood ratio tests presented in the Supplementary Material cover the general case of more than two variance–covariance matrices. This approach can also be applied to higher dimensional variance–covariance matrices. The two-dimensional case presented here is determined from the interaction between an *a priori* selected seed and any other voxel in the brain. A more general, multidimensional case could include more than one pivotal seed. In such a case, we could test not only for seed-based connectivity, but also for network-based connectivity patterns determined by any *a priori* network. Our methodology could also be potentially extended to longitudinal data. In this case, the expressions for the likelihood ratio tests presented in the Supplementary Material would need to be modified to cover suitable within-subject correlations patterns (e.g., paired samples). While, to the best of our knowledge, the requisite theory has not yet been developed and is beyond the scope of this manuscript, we believe that such a theoretical formulation could be realized.^39^ Although we have used a relatively large sample size in this study, our methodology can be adapted to increase the statistical power in the case of smaller sample size.^40^ Specifically, it was shown by Rayner *et al.*^40^ that corrected hierarchical tests (resulting from multiplying the tests T_i_ by certain correcting factors) produce actual sizes closer to the nominal sizes for small samples. For larger sample sizes, the hierarchical tests have desirable power properties, as the proposed correcting factor tends to 1 for large sample size. Our hierarchical testing approach can also be used to assess the equality/proportionality of covariance matrices to use the most appropriate model for multivariate tests comparing mean signals, as well as for selection of optimal discriminant analysis functions. Finally, this framework may be generalized to other connectivity measures, such as functional connectivity derived from blood oxygen level-dependent functional MRI, potentially providing unique insights into disease-related alterations in the functional architecture of the brain.

## Figures and Tables

**Table 1:** Summary of subject characteristics

	*All subjects*	*Aβ_L_*	*Aβ_H_*
Sample size	276	137	139
SUVR_ROI_	1.26±0.27	1.03±0.08	1.50±0.16
Age	72.83±7.96	71.47±8.60	74.22±7.02
Gender (F/M)	119/157	61/78	58/79
APOE ɛ4 (carrier/non-carrier)	132/144	35/102	98/41
MMSE	27.34±3.25	28.22±2.44	26.45±3.71

Abbreviations: A*β*_H_, Amyloid-High; A*β*_L_, Amyloid-Low; MMSE, mini-mental state exam; APOE, apolipoprotein E; ROI, region of interest; SUVR, standardized uptake value ratio.

## Notes

FC, AC, and APZ are employees of Biospective. APZ has received shares from Biospective. ACE and BJB have received compensation and shares from Biospective.
