# Supplementary material for: Hierarchical multivariate covariance analysis of metabolic connectivity
Source: J Cereb Blood Flow Metab. 2014 Oct 8;34(12):1936–43. doi: 10.1038/jcbfm.2014.165 (PMC4269748; doi:10.1038/jcbfm.2014.165)
Supplement: Supplementary Information [file jcbfm2014165x1.pdf]

Supplementary Material

$$T_1 = (n-g)\log\left(\frac{|\Omega_0|}{|\Omega_1|}\right) - 4\sum_{j=2}^{g}(n_j - 1)\log(c_j),\ df_1 = g\text{-}1,$$

$$T_2 = (n-g)\log\left(\frac{|\Omega_1|}{|\Omega_2|}\right) - 2\sum_{j=2}^{g}(n_j - 1)\left(\log(c_{1j}c_{2j}) - 2\log(c_j)\right),\ df_2 = 2(g\text{-}1),$$

$$T_3 = \sum_{j=1}^{g}(n_j - 1)\log\left(\frac{|\Omega_2|}{|S_j|}\right) + 2\sum_{j=2}^{g}(n_j - 1)\log(c_{1j}c_{2j}),\ df_3 = g\text{-}1,$$

$$T_0 = \sum_{j=1}^{g}(n_j - 1)\log\left(\frac{|\Omega_0|}{|S_j|}\right),\ df_0 = 3(g\text{-}1)$$

where

S_j denotes the sample covariance matrix corresponding to the group j $(j=1,...,g)$,

estimated with sample size equal to n_j, $n = \sum_{j=1}^{g} n_j$ and $\Omega_0 = \frac{\sum_{j=1}^{g}(n_j - 1)S_j}{n-g}$.

The maximum likelihood estimates of the matrix Ω_1 and the constants c_j are solutions of the system of equations:

$$\Omega_1 = \sum_{j=1}^{g}\frac{(n_j - 1)S_j}{(n-s)c_j^2},$$

$$c_j = \sqrt{\frac{1}{2}tr\left((\Omega_1)^{-1}S_j\right)}$$

which can be readily solved by iteration.

The maximum likelihood estimates of the matrix Ω_2 and the constants c_{1j}, c_{2j} are solutions of the system of equations:

$$\Omega_2 = \sum_{j=1}^{g} \frac{(n_j - 1) C_j^{-1} S_j C_j^{-1}}{n - s}$$

$$c_{tj} = \sum_{r=1}^{2} \frac{(\Omega_2)_{rt}^{-1} (S_j)_{rt}}{c_{rj}}, t = 1,2, j = 2,\ldots, g$$

,

where $C_j = \begin{pmatrix} c_{1j} & 0 \\ 0 & c_{2j} \end{pmatrix}$, and *(.)*$_{rt}$ denotes the matrix element corresponding to the row *r* and column *t.* This system can also be solved by iteration.

Supplementary Figures

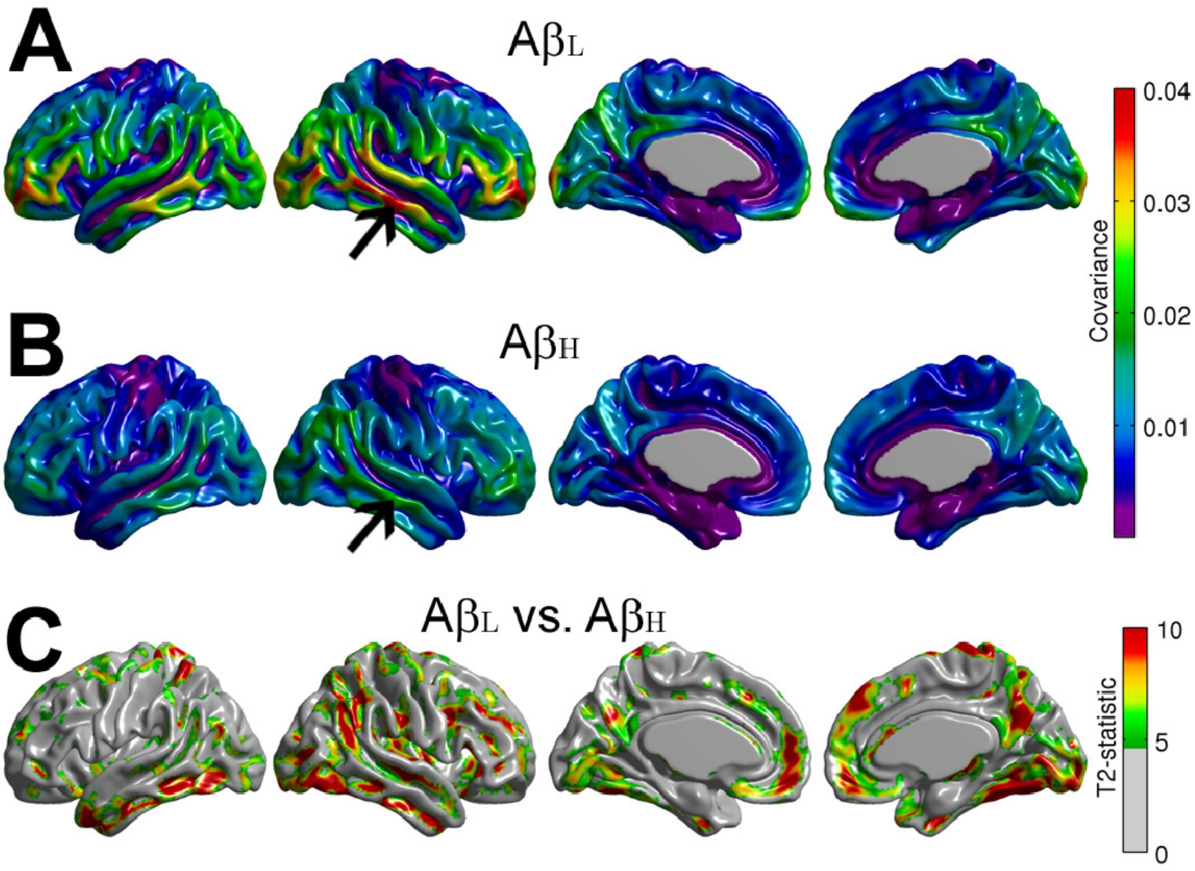

Supplementary Figure 1. Seed-based covariance maps for the right middle temporal gyrus. Maps are provided for the $A\beta_L$ group (A), $A\beta_H$ group (B), as well as FDR-thresholded T_2-statistic for the $A\beta_L$ vs. $A\beta_H$ group comparison (C), which shows several regions of statistically significant differences, particularly between the seed and bilateral inferior temporal gyrus, fusiform gyrus, and precuneus. The arrows indicate the seed region.
